# Altered Expression of Hematopoiesis Regulatory Molecules in Lipopolysaccharide-Induced Bone Marrow Mesenchymal Stem Cells of Patients with Aplastic Anemia

**DOI:** 10.1155/2018/6901761

**Published:** 2018-10-17

**Authors:** Chandra Prakash Chaturvedi, Naresh Kumar Tripathy, Ekta Minocha, Akhilesh Sharma, Khaliqur Rahman, Soniya Nityanand

**Affiliations:** Stem Cell Research Facility, Department of Hematology, Sanjay Gandhi Postgraduate Institute of Medical Sciences (SGPGIMS), Raebareli Road, Lucknow 226014, India

## Abstract

We have investigated the expression of RNA transcripts of hematopoiesis regulatory molecules, viz., macrophage inflammatory protein (MIP)-1*α*, tumor necrosis factor (TNF)-*α*, granulocyte colony-stimulating factor (G-CSF), stromal cell-derived factor (SDF)-1*α*, stem cell factor (SCF), and transforming growth factor (TGF)-*β* in lipopolysaccharide-induced bone marrow mesenchymal stem cells (BM-MSCs) and levels of their soluble forms in the culture supernatants of BM-MSCs and BM plasma of patients with acquired aplastic anemia (AA) (*n* = 29) and controls (*n* = 29). The BM-MSCs of AA patients as compared to controls had markedly lower expression of MIP-1*α* transcripts (*p* < 0.001), higher expression of TNF-*α* (*p* < 0.001), G-CSF (*p* < 0.001), and SDF-1*α* (*p* < 0.01) transcripts, and no difference in the expression of SCF and TGF-*β* transcripts. The culture supernatants of BM-MSCs and BM plasma of AA patients in comparison to controls also had lower levels of MIP-1*α* (*p* < 0.01 and *p* < 0.001, respectively) and higher levels of TNF-*α* (*p* < 0.05 for both) and G-CSF (*p* < 0.05 and *p* < 0.001, respectively) but with no difference in the levels of SDF-1*α* and SCF. The levels of TGF-*β* were although similar in culture supernatants of BM-MSCs of both the groups, but they were significantly lower in BM plasma of the patients than controls (*p* < 0.001). Our data shows that BM-MSCs of AA patients have altered expression of hematopoiesis regulatory molecules suggesting that they may have a role in the pathogenesis of the disease.

## 1. Introduction

Acquired aplastic anemia (AA) is a state of bone marrow failure characterized by peripheral pancytopenia and a hypoplastic bone marrow (BM) where the normal hematopoietic cells are replaced by fat cells. The majority of the cases of AA are idiopathic where the cause is not known and most studies have focused on intrinsic hematopoietic stem cell defect or immune-mediated destruction of hematopoietic stem cells (HSCs) in the pathogenesis of AA, but a precise pathogenic mechanism of the disease remains unclear till now [1, 2].

BM mesenchymal stem cells (BM-MSCs) and their differentiated cells constitute the hematopoietic niche in the BM and have a vital role in maintaining long-term hematopoiesis [2].We and other groups have reported an increased adipogenic and/or decreased osteogenic differentiation potential of BM-MSCs in AA patients [3–5]. An increase in the adipogenesis and a decrease in the osteogenesis in the BM negatively affect hematopoiesis [6, 7], and hence, these alterations in the differentiation potential of BM-MSCs of AA patients may contribute to the deficient hematopoiesis in AA. In addition, using crossover coculture experiments, it has also been demonstrated that BM-MSCs of AA patients have deficient hematopoiesis-supportive activity [8, 9]. Since BM-MSCs interact with HSCs mainly by secreting paracrine molecules including cytokines, chemokines, and growth factors to maintain hematopoietic homeostasis [2, 10], hence our hypothesis is that an altered expression of hematopoietic regulatory molecules may be involved in mediating these functional abnormalities of BM-MSCs in AA.

Therefore, the aim of the present study was to study the expression of hematopoietic regulatory molecules including macrophage inflammatory protein (MIP)-1*α*, tumor necrosis factor (TNF)-*α*, granulocyte colony-stimulating factor (G-CSF), stromal cell-derived factor (SDF)-1*α*, stem cell factor (SCF), and transforming growth factor (TGF)-*β* by BM-MSCs of AA patients.

## 2. Materials and Methods

### 2.1. Subjects

Twenty-nine AA patients (12 females, 17 males) with a mean age of 32.86 ± 16.43 years (range 12–64 years) and the same number of age- and sex-matched controls were recruited in the study. Diagnosis and grading of severity of AA was done as per published criteria [11]. Controls included 10 healthy donors and 19 patients with immune thrombocytopenic purpura who had undergone a diagnostic BM testing but had normal marrow. After informed consent, 5 ml of BM from the posterior superior iliac crest of each subject was aspirated into heparinized tube for isolation and culture of BM-MSC.

### 2.2. Isolation, Culture, and Immunophenotypic Characterization of BM-MSCs

The BM-MSCs were isolated, cultured, and characterized as described earlier [3]. Briefly, mononuclear cells obtained from the BM aspirates by density gradient centrifugation were cultured in 25 cm^2^ flasks (BD Biosciences, USA) at 37°C in 5% CO_2_ using 5 ml of complete culture media consisting of *α*-MEM, 1% GlutaMAX, 16.5% fetal bovine serum, 100 U/ml penicillin, and 100 *µ*g/ml streptomycin (all from Gibco and Thermo Fisher Scientific, USA). After 48 hours, nonadherent cells were removed and medium was replaced. When culture reached 70–80% confluency, adherent cells were harvested using 0.05% trypsin (Gibco) and replated for further expansion. The cells of the 3^rd^ passage were used in all the experiments.

For immunophenotypic characterization, the BM-MSCs were stained by incubating the cells for 30 minutes with the following preconjugated antibodies: CD73 (PE), CD90 (PE), CD105 (PE), CD166 (PE), CD34 (FITC), CD45 (FITC), HLA-DR (FITC), and CD13 (PE) (all from Serotec, (http://www.abdserotec.com)). Cells stained with isotype-matched antibodies served as controls. After washing, the cells were acquired on a BD FACS-Canto flow cytometer (BD Biosciences, San Jose, CA, USA) and the data analysis was done using the FACS express software.

### 2.3. Differentiation of BM-MSCs into Adipocytic and Osteocytic Lineages

Differentiation of BM-MSCs into adipocytic and osteocytic cells was carried out using the adipogenesis and osteogenesis kits, respectively (both from Gibco, Gaithersburg, MD, USA) as per the manufacturer's instructions and already reported by us [3]. For adipogenic and osteogenic differentiation, the cells were fixed and stained with oil red O stain after 18 days, and with alizarin red stain after 21 days of incubation in the differentiation medium, respectively.

### 2.4. RNA Extraction and Quantitative Real-Time PCR (qRT-PCR)

BM-MSCs obtained from the 3^rd^ passage semiconfluent cultures were cultured for further 24 hours in plain alpha MEM medium (Gibco) supplemented with 500 ng/ml lipopolysaccharide (LPS; Sigma-Aldrich, St. Louis, Missouri, USA) and trypsinized after 24 hours and total RNA was extracted using TRIzol reagent (Invitrogen, Waltham, Massachusetts, USA). The cDNA synthesis and quantitative real-time PCR were performed as previously established in the lab [3]. The fold expression change was calculated using 2^−ΔΔCt^ method. The oligonucleotides used as primers (from MWG Biotech Pvt. Limited, Bangalore, India) for RT-qPCR are given in Table 1.

### 2.5. Quantization of Hematopoietic Regulatory Cytokines in BM-MSC Culture Supernatants and BM Plasma

To prepare culture supernatant, the 3^rd^ passage BM-MSCs were cultured for 24 hours in 2.5 ml of plain alpha MEM medium (Gibco) containing 500 ng/ml of LPS. The culture supernatant of these cultures was harvested and centrifuged at 1000*g* for 10 minutes to remove any cellular debris. The BM plasma was collected by centrifuging the marrow aspirate at 1000*g* for 10 minutes. The culture supernatant and BM plasma were stored at −20°C until use. Quantitative estimation of G-CSF, SCF, SDF-1, MIP-1*α*, TNF-*α*, and TGF-*β* in the culture supernatants and concomitantly in the BM plasma of each subject was performed by ELISA kits (R&D Systems, Minneapolis, MN, USA), as per manufacturer's instructions.

### 2.6. Statistical Analysis

The results were calculated as mean ± SE. The statistical significance was determined by Student's *t*-test using the GraphPad Prism Software Version 5 (GraphPad, San Diego, CA, USA). The *p* values < 0.05 were considered to be statistically significant.

## 3. Results

### 3.1. Morphology and Immunophenotypic Characterization

**Figure 1:**
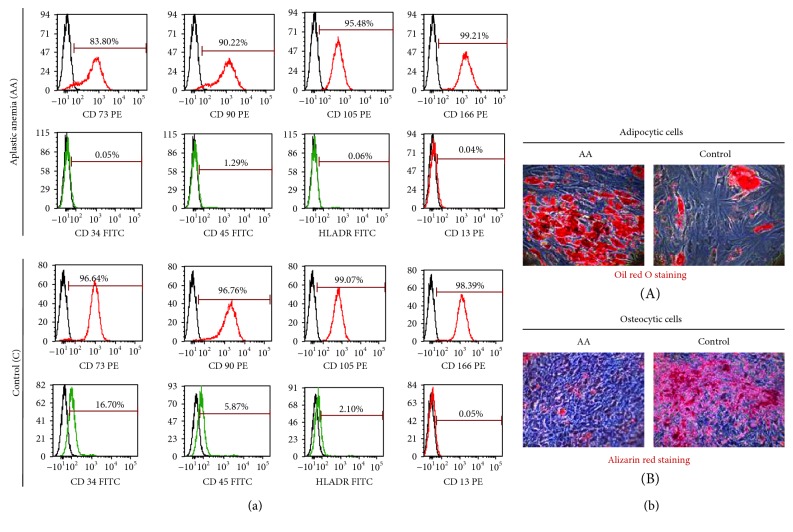
Immunophenotypic and differentiation characterization of BM-MSCs of aplastic anemia (AA) patients. (a) Representative flow cytometric histograms showing immunophenotype of BM-MSCs of AA patients and controls. (b) (A) Oil red O staining of adipocytes differentiated from BM-MSCs of AA patients and controls. (b) (B) Alizarin red staining of osteocytes differentiated from BM-MSCs of AA patients and controls.

The BM-MSCs of AA patients showed fibroblast-like spindle-shaped morphology similar to BM-MSCs of controls. The flow cytometric analysis of their phenotypic markers showed that BM-MSCs of AA patients as well as of controls expressed comparable levels of MSC markers CD73, CD89, CD105, and CD166 and lacked the expression of CD13, CD34, CD45, and HLA-DR (Figure 1(a)).

### 3.2. Adipogenic and Osteogenic Differentiation of BM-MSCs

The oil red O staining of the adipocytes differentiated from AA BM-MSCs showed higher density and larger size of lipid droplets suggesting a higher adipogenic potential of AA BM-MSCs as compared to control BM-MSCs (Figure 1(b)). However, BM-MSCs of the AA patients showed a lesser osteogenic potential as compared to the control MSCs as reveled by alizarin red staining (Figure 1(b)).

### 3.3. Expression of Hematopoiesis Regulatory Genes in BM-MSCs

**Figure 2:**
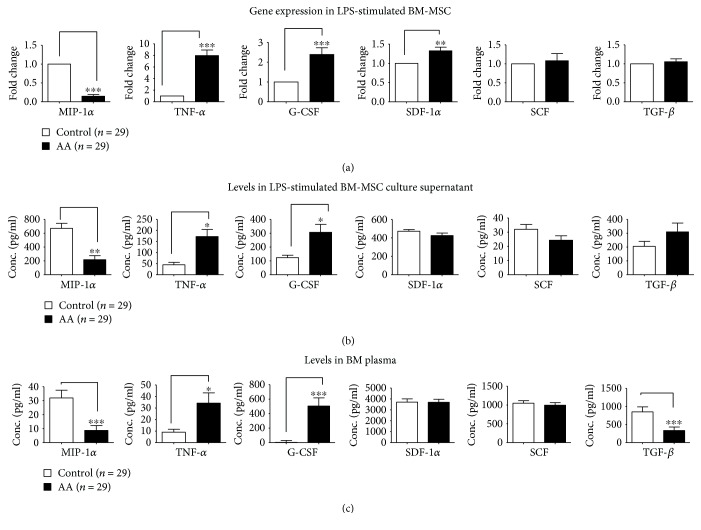
Expression of the hematopoietic regulatory molecules: MIP-1*α*, TNF-*α*, G-CSF, SDF-1*α*, SCF, and TGF-*β*1. (a) Relative fold changes in mRNA expression of hematopoietic regulatory cytokine genes in AA BM-MSCs as compared to control BM-MSCs by qRT-PCR after LPS stimulation. (b) Levels of the hematopoietic regulatory molecules in culture supernatants of LPS-stimulated AA and control BM-MSCs as detected by ELISA. (c) Levels of the hematopoietic regulatory molecules in the BM plasma of AA patients and controls as detected by ELISA. Values represent mean ± SE. ^∗∗∗^*p* < 0.001, ^∗∗^*p* < 0.01, and ^∗^*p* < 0.05 between AA patients and controls.

The BM-MSC of AA patients (*n* = 29) in comparison to controls (*n* = 29) showed no difference in the constitutive expression of transcripts of MIP-1*α*, TNF-*α*, TGF-*β*1, G-CSF, SDF-1*α*, and SCF. However, following LPS stimulation, the transcript levels of MIP-1*α* were dramatically lower (fold decrease: 7.1; *p* < 0.001) while those of TNF-*α* (fold increase: 7.0; *p* < 0.001), G-CSF (fold increase: 2.38; *p* < 0.001), and SDF-1*α* (fold increase: 1.36; *p* < 0.01) were significantly higher with no difference in the levels of the SCF and TGF-*β*1 transcripts, between patients and controls (Figure 2(a)).

### 3.4. Secretion of Hematopoiesis Regulatory Molecules by AA BM-MSCs

The culture supernatants of BM-MSCs of AA patients (*n* = 29) compared to controls (*n* = 29) showed no difference in the levels of MIP-1*α*, TNF-*α*, TGF-*β*1, G-CSF, SDF-1*α*, and SCF levels. However, following LPS stimulation, the culture supernatants of BM-MSCs of the patients compared to controls also had significantly lower levels of MIP-1*α* (216.7 ± 58.72 vs 670.1 ± 72.85; *p* < 0.01) and higher levels of TNF-*α* (172.32 ± 32.05 vs 44.76 ± 10.89 *p* < 0.05), and G-CSF (306.8 ± 56.90 vs 123.2 ± 17.68; *p* < 0.05) with no difference in the levels of SCF (24.32 ± 3.09 vs 31.98 ± 3.45; *p* > 0.05) and TGF-*β*1 (310.0 ± 63.68 vs 204.70 ± 36.37; *p* > 0.05)(Figure 2(b)).

### 3.5. Levels of Hematopoiesis Regulatory Molecules in BM Plasma

The BM plasma of AA patients (*n* = 29) in comparison to that of controls (*n* = 29) also showed significantly lower levels of MIP-1*α* (8.70 ± 3.51 vs 31.91 ± 5.58; *p* < 0.001) and higher levels of TNF-*α* (34.26 ± 8.87 vs 9.07 ± 2.47; *p* < 0.05), and G-CSF (505.32 ± 112.80 vs 3.48 ± 5.99; *p* < 0.001), with no difference in the levels of SCF (996.20 ± 67.68 vs 1050 ± 64.70; *p* > 0.05) and SDF-1*α* (3695.0 ± 268.4 vs 3706.0 ± 296.0; *p* > 0.05). However, the levels of TGF-*β*1 were significantly lower in BM plasma of patients than controls (335.2 ± 97.07 vs 850.7 ± 139; *p* < 0.001) (Figure 2(c)).

## 4. Discussion

We observed that BM-MSCs of AA patients although have a morphology and phenotype similar to controls but have a higher adipogenic and lower osteogenic differentiation potential, which is consistent with previous reports of our and other groups [3–5]. To study the role of BM-MSC derived hematopoiesis regulatory molecules in AA, we initially evaluated the expression of MIP-1*α*, TNF-*α*, G-CSF, SDF-1*α*, SCF, and TGF-*β*1 transcripts in BM-MSCs of AA patients and controls as well as levels of soluble forms of these hematopoiesis regulatory molecules in the culture supernatants of BM-MSCs of both the groups under constitutive conditions but observed no significant difference between the two groups either in the expression of the transcripts of these molecules or in their soluble protein levels in the cell culture supernatants. Therefore, we measured these hematopoiesis regulatory cytokines following stimulation of BM-MSCs with LPS, which is a potent stimulant of these cells and induces their activation via binding to their Toll-like receptors [12, 13]. The LPS-stimulated BM-MSCs of AA patients as compared to controls exhibited markedly lower expression of MIP-1*α* and higher expression of TNF-*α*, G-CSF, and SDF-1*α* transcripts as well as lower MIP-1*α* and higher TNF-*α* and G-CSF levels in the culture supernatants with no difference in the levels of SDF-1*α*, SCF, and TGF-*β*1. The BM plasma of AA patients also had alterations in the levels of these hematopoiesis regulatory molecules corroborating with the alterations in the levels in the culture supernatants, except for TGF-*β*1 where the levels were reduced in the BM plasma though the levels were comparable in the culture supernatants of patients and controls. Although a few sporadic reports are available in literature on these hematopoiesis regulatory molecules in bone marrow mononuclear cells of AA patients, but to the best of our knowledge, this is the first study on transcripts of these molecules in BM-MSCs and their protein levels in the culture supernatant of BM-MSCs as well as in BM plasma of AA patients.

There is no previous report on MIP-1*α* transcripts or proteins in BM-MSCs of patients with AA. However, a single report on long-term marrow cultures of AA patients has shown elevated levels of MIP-1*α* in the baseline culture supernatants of 14 of 20 AA patients, and following IL-1*α* stimulation of the cultures, the levels further increased in 7 patients but markedly decreased in remaining 7 patients as observed by us in the present study [14]. MIP-1*α* is reported to suppress proliferation of primitive HSCs probably to maintain their quiescence while promoting proliferation of mature HSCs highlighting its bidirectional role in hematopoiesis [15]. In addition, MIP-1*α* is also important in mediating interaction between MSCs and HSCs or immune cells of the BM to modulate their functional properties [16]. Hence, our observation on the almost absent expression of MIP-1*α* in BM-MSCs and the corresponding decrease in its levels in BM plasma of AA patients is likely to impair the cell-cell interaction of HSCs with MSCs and in turn the hematopoiesis supporting functions of BM-MSCs.

TNF-*α* is reported to cause bone marrow aplasia in AA by inducing apoptotic cell death of HSCs through Fas-Fas ligand and/or TNF-related apoptosis-inducing ligand pathways [17]. Increased levels of TNF-*α* have been reported in AA, and T-cells present in marrow of the patients are considered to be the main source of TNF-*α* [18, 19]. Our demonstration of higher expression of TNF-*α* at transcript as well as protein levels in BM-MSCs of patients with AA is the first report showing that BM-MSCs are also an important source of TNF-*α* in BM of AA patients. Our previous study showing increased levels of TNF-*α* in BM plasma but no expression in marrow T-cells also supports our current observation [20].

The BM-MSCs of AA patients had higher expression of G-CSF transcripts with corresponding higher levels in culture supernatants. It is an important member of the hematopoietic cytokine family secreted by stromal and other cell types. Similar to our observation, elevated levels of G-CSF transcripts have been reported in BM stromal cells and their culture supernatants [21, 22]. On the one hand, increased expression of G-CSF is considered to represent a compensatory supraphysiological response of BM-MSCs to boost reduced hematopoiesis in AA. On the other hand, an inverse correlation of higher G-CSF levels with numbers of myeloid cells has been reported in AA patients. However, after BM transplantation or immunosuppressive therapy, the G-CSF levels have been shown to return to normal levels in AA patients, which together suggest an antimyelopoietic effect of G-CSF in AA [23, 24]. The expression of SDF-1*α* transcripts in BM-MSCs of patients with AA although was statistically higher as compared to controls, but the levels in the culture supernatant were similar to controls. The levels of SCF, which is solely produced by marrow stromal cells, were comparable to controls at both the gene and protein levels. Data of previous studies on SDF-1*α* and SCF in marrow stromal cells corroborates with our observation [25, 26].

In order to ascertain the alterations in these hematopoiesis regulatory molecules *in vivo* in the marrow microenvironment as well, we analyzed their levels in BM plasma of patients with AA. It was observed that similar to their levels in culture supernatant of BM-MSCs, the BM plasma of AA patients as compared to controls had lower levels of MIP-1*α* and higher levels of TNF-*α* and G-CSF with no difference in the levels of SCF and SDF-1*α*. The alterations in the BM plasma levels of these cytokines also corroborated with the levels of the mRNA transcripts in LPS-stimulated BM-MSCs of AA patients, but since there is widespread adipogenesis in the marrow of AA patients, hence BM adipocytes and other cell-like macrophages/T-cells may also have an important contribution to the alterations of these cytokines in BM plasma of AA patients. The levels of TGF-*β*1 were significantly lower in BM plasma of the patients than controls, unlike the comparable expression in the culture supernatants of BM-MSCs implying a deficient production of this cytokine by other marrow cell-like T-cells and/or macrophages. Similar to our observation, lower levels of TGF-*β*1 have been reported previously in long-term marrow cultures of AA patients [27]. Since one of the main functions of TGF-*β*1 is to mediate expansion of regulatory T-cells, the reduced level of this cytokine may be responsible for the decreased numbers of Tregs reported in the marrow of AA patients [5]. Future studies exploring the role of MIP-1*α*, TNF-*α*, and G-CSF in hematopoietic and immunomodulatory functions of BM-MSCs of AA would be important for better understanding the pathophysiology of the disease.

## 5. Conclusion

Our data shows that BM-MSCs of AA patients have markedly lower expression of MIP-1*α* and higher expression of TNF-*α* and G-CSF transcripts with corresponding alteration of their levels in the culture supernatant as well as in BM plasma, collectively highlighting an abnormality of BM-MSCs in AA.

## Figures and Tables

**Table 1:** List of primer sequences used for RT-qPCR.

SDF-1 (CXCL12)	
Forward	5′-ATGAACGCCAAGGTCGTGGTC-3′
Reverse	5′-GGTCTGTTGTGCTTACTTGTTT-3′
SCF (c-kit ligand)	
Forward	5′-ACT GAC TCT GGA ATC TTT CTC AGG-3′
Reverse	5′-GAT GTT TTG CCA AGT CAT TGT TGG-3′
G-CSF	
Forward	5′-TCTGAGTTTCATTCTCCTGCCTG-3′
Reverse	5′-ATTTACCTATCTACCTCCCAGTCCAG-3′
TNF-*α*	
Forward	5′CCCAGGGACCTCTCTCTAATCA-3′
Reverse	5′AGCTGCCCCTCAGCTTGAG-3′
TGF-*β*1	
Forward	5′-AGTGGACATCAACGGGTTCAG-3′
Reverse	5′-CATGAGAAGCAGGAAAGGCC-3′
MIP-1*α* (CCL3)	
Forward	5′-TGCCTGCTGCTTCTCCTACA-3′
Reverse	T5′-TGGACCCAGGTCTCTTTGGA-3′
GAPDH	
Forward	5′-CCCCTTCATTGACCTCAACTAC-3′
Reverse	5′GATGACAAGCTTCCCGTTCTC-3′

## Data Availability

The data used to support the findings of this study are included within the article. Any additional information about the data used to support the findings of this study are available from the corresponding author upon request.
